# Maternal mental health concerns during the COVID-19 pandemic in Iran: a content analysis study

**DOI:** 10.1186/s12978-023-01645-5

**Published:** 2023-07-11

**Authors:** Pouran Mokhtari Zanjani, Najmeh Tehranian, Nasrin Changizi, Eesa Mohammadi, Banafsheh Farzinrad, Anoshirvan Kazemnejad

**Affiliations:** 1 Department of Reproductive Health and Midwifery, Faculty of Medical Sciences, Tarbiat Modares University, Tehran, Iran; 2 Maternal, Fetal and Neonatal Research Center, Tehran University of Medical Sciences, Tehran, Iran; 3 Department of Nursing, Faculty of Medical Sciences, Tarbiat Modares University, Tehran, Iran; 4 School of Behavioral Sciences and Mental Health, Iran Medical Science University, Tehran, Iran; 5 Department of Biostatistics, Faculty of Medical Sciences, Tarbiat Modares University, Tehran, Iran

**Keywords:** Mental health, COVID-19 pandemic, Maternal

## Abstract

**Background:**

As crises occur, attention to physical health usually becomes more than mental health, neglecting mental health, especially in some vulnerable groups such as pregnant women and new mothers, can have adverse consequences. Therefore, it is imperative to recognize and comprehend their mental health needs, particularly during critical situations such as the recent COVID-19 pandemic. The aim of this study was to explain the understanding and experience of mental health concerns faced by pregnant and postpartum women during this pandemic.

**Methods:**

This qualitative study was conducted from March 2021 to November 2021 in Iran. Data were collected using semi-structured in-depth interviews to understand mental health concerns in pregnancy and the postpartum period during the COVID-19 pandemic. Twenty-five purposefully retrieved and participated in the study. Due to the prevalence of coronavirus, most of the participants preferred tele interviews. When data saturation was achieved, the data were codified manually and analyzed using Graneheim and Lundman’s approach 2004.

**Results:**

Based on the content analysis of the interviews, 2 main themes, 8 categories 23 subcategories were identified. The identified themes were as follows: (1) Maternal mental health threats and (2) Inadequate access to the required information.

**Conclusion:**

The results of this study showed that the main concern and fear of pregnant and postpartum women in the COVID-19 pandemic was the fear of the possibility of death for themselves and/or their fetus/infant. The knowledge that was obtained from the understanding of pregnant women and new mothers about the mental health concerns during the COVID-19 pandemic can help managers in planning to improve and promote women's mental health, especially in critical situations.

## Background

According to the definition of the World Health Organization (WHO) "Maternal mental health is a state of well-being in which the mother realizes her own abilities, can cope with the normal stresses of life, can work productively and fruitfully, and is able to make a contribution to her community" [1]. During pregnancy and postpartum various factors affect maternal mental health, one of which is the occurrence of stressful events [2]. The most recent stressful crisis is the COVID-19 disease since December 31, 2019 [3]. The COVID-19 pandemic has severely disrupted people's lives and has had a tremendous and deep effect on human life. The measures taken to prevent this disease, such as various forms of physical distancing, quarantine, have caused a mental health crisis around the world [4–6]. However, the severity of the problems caused by the pandemic has not been the same for all people, some groups are more vulnerable such as women during pregnancy and postpartum due to their special physical and psychological needs. During this period, they faced new challenges and concerns such as fear of getting the infection, vertical transmission to the fetus, limited access to pre-and post-natal care, reduced social support, and the existence of false information [7–14]. There is evidence of the long-term mental health effects of previous pandemics and disasters [15–17]. Evidence shows that there has been a significant increase in various mental health problems among the general population and vulnerable groups due to the COVID-19 pandemic [6]. It is obvious that the occurrence of stress, anxiety, post-traumatic stress, etc. disrupts maternal mental health, and causes a range of physical, mental, and behavioral disorders in both mothers and infants [18–23]. Several qualitative studies in different countries such as Australia, Turkey, Ireland, Canada, and Iran have also investigated various aspects of COVID-19 in mothers [24–30]. Given the extent of the COVID-19 pandemic, it is predicted that people's mental health will be affected by its direct and indirect psychological effects both presently and in the future [4, 31, 32]. As the world slowly regains control of COVID-19, it is timely and essential to explore the impact of COVID-19 on mental health, especially among vulnerable populations, around the world [33]. To deeply understand the concerns of Iranian healthy and the get infected women during pregnancy and postpartum, this study was designed and implemented with a qualitative approach and provided valuable insights into the experiences of these women.

## Methods

### Aim

The purpose of this study was to explain the understanding and experience of pregnant and postpartum women's mental health concerns during the COVID-19 pandemic.

### Design

The current qualitative study was conducted using the conventional content analysis approach to explain the women in pregnancy and postpartum period mental health concerns during COVID-19. Conventional content analysis is a qualitative method that allows the researcher to discover and explain the perceptions and experiences of individuals in a specific social and cultural context [34].

### Setting

This study was performed in comprehensive health centers and a referral hospital university affiliated with Zanjan University of Medical Sciences, Iran.

## Participant

### Participant inclusion criteria

All pregnant women of any gestational age, all mothers in the first 6-month postpartum period, who accepted the participation and were willing to participate in this research were included and enrolled in the study. A total of twenty-five people fourteen during pregnancy and eleven in postpartum) participated in this study.

For all participants (women during pregnancy and postpartum period) the following criteria were met: willingness to participate in the study, having Iranian nationality, and being able to communicate and share their experiences. To maximize diversity in terms of age, education, gestational age, postpartum time, and having coronavirus disease (COVID-19) (during pregnancy, postpartum, pre-pregnancy) and spouse with coronavirus 2019, we used purposive sampling with maximum variety.

### Data collection procedure

Semi-structured, in-depth interviews were conducted by the first author (PMZ, MSc, PhD student, female) from March 2021 to November 2021. In order to maintain the health of the participants and due to the reluctance of the participants for face-to-face interviews (due to fear of contracting the coronavirus), most of the interviews were done by a mobile phone with voice recording software was used. At the beginning of each interview, the researcher stated the purpose of the research, made the necessary arrangements in terms of time and type of interview (phone or face-to-face), and obtained permission to record the interview from the participants, then the questions of each group were asked (The interview guide was presented in Table 1). After receiving the answer, probing questions such as: Can you explain more about this? What do you mean? Can you give an example were asked, and finally, open-ended questions were asked to identify the specific needs of the participants—for example: Is there a concern or need that I did not ask you and you want to explain? Each interview lasted 24 to 65 min, but one interview lasted 120 min because the interviewee was multiple concerns about being infected with the coronavirus. Interviews continued until data saturation. The collection continued until data saturation (i.e., no new code was extracted following the interviews). Data saturation occurred after interview 20 and was continued for up to 5 more people to be sure. to conduct this research, permission was first obtained from the ethics committee of Tarbiat Modares University. then, written consent forms were designed, although, for the telephone interviews, the contents of the consent were read orally to the participants. Before starting the interview, permission to record the conversations and its purpose was explained to them, and the consent to record the conversations was obtained. The time and type of interview (telephone or face-to-face) were determined with the consent of the participants and based on their choice. Participants were assured that their statements would remain confidential. So that their data is analyzed and reported anonymously using management numbers. Furthermore, Participants were assured that they would be free to withdraw from the study at any stage.

**Table 1:** The interview guide

The interview Questions (For women during pregnancy and postpartum period)
Tell me how do you feel about this pregnancy?
How do you feel about getting pregnant during covid-19 pandemic?
Tell me about your feelings and interests in life and daily activities?
What information do you need about your pregnancy?
What information do you need about your pregnancy during this pandemic?
What information and knowledge do you need about the fetus?
Talk about your spouse's support and attention during this pregnancy? How is it? How do you feel about supporting your spouse?
Did you expect/receive support from others during this pregnancy? What support and attention (if any)? Please explain
Can you tell me about your feelings and mental image of your body and physical condition that changed during pregnancy, how do you feel about it?
What is your main concern in this situation?
Tell me how you feel about the delivery process
Explain what type of delivery did you choose?
How do you feel about giving birth in a COVID-19 pandemic?
What are your concerns about the fetus/baby regarding the condition of the corona? Please explain
How did you feel about your pregnancy during and after the birth? Has it changed or been like your pregnancy? Explain if possible
Tell me about your feelings and relationship with your baby after giving birth in the hospital and then at home
What information did you need and search for after giving birth?
Please explain about your doctor/midwife paying attention during your pregnancy. What did you expect from them? Were your expectations met?
Please explain about your doctor/midwife pay attention during your labor and after birth. What did you expect from them? Were your expectations met?
Can you tell me about your feelings and mental image of your body and physical condition that changed after birth, how do you feel about it?
Is there another feeling, concern, need that I have not asked and you want to say?

### Data analysis

Data collection and analysis were performed simultaneously [35]. Data analysis was performed using content analysis. Conventional content analysis is a systematic and objective way to describe phenomena [36, 37], that leads to a valid inference from the data and is suitable for examining people's experiences and opinions on the topic of interest [38], and its primary purpose is a conceptual description of the phenomenon [39]. In the current study, “Qualitative content analysis based on Lundman, Graneheim 2004” five steps were used. It means that Data collection and analysis were done simultaneously, immediately after each interview, it was written down word by word, and the text was reviewed and read several times with the aim of obtaining a general understanding of the content [35].

During the data analysis process, at first, the whole text of each interview was considered as an analysis unit whose meaning units were groups of words or sentences which were relevant to the study topic or question. Next, the primary codes that were the result of condensed meaning units were extracted and were named and classified based on their similarities. With the progress or increase in the number of interviews and the extraction of more codes and subcategories, through ongoing comparison of them based on similarities, differences, and proportions, abstraction was performed. Finally, as we approached saturation, we classified and then finally extracted themes which we did it manually.

An example of the formation of a theme is given in Table 2.

**Table 2:** An example of the process of extracting a theme from semantic units, codes

Semantic units	Primary codes	Subcategory	category	category
… It was constantly said on TV that someone got corona and died of it and…..I would definitely die of corona disease because I was pregnant	Hearing the death of people repeatedly	Fear of her own death	Fear of death	maternal mental health threats
…When I hear the news of the death of people who are infected, I am really stressed and I am afraid that I will die too…				
…. I am sure I will die because I am pregnant I know I will die ….	Increased fear of dying due to pregnancy			
So far, some pregnant have been infected and died … I was also afraid to die…				
In my previous pregnancy, a woman's baby was stillborn. Sometimes I worry that my baby will die during childbirth or die in my womb! When I worry like this, I go to the doctor, and when I hear the baby's heartbeat, I calm down	The experience of hearing the death of another's fetus and the fear of its occurrence	Fear of fetal death		
It happened to one of my colleagues that the umbilical cord got wrapped around his baby's neck and died, I'm afraid it will happen to me and my baby will die				
My stress is so that nothing happens to my baby in these 9 months and I spend this period healthy and my baby is born healthy and alive	Fear of having an unfortunate event and losing the fetus during pregnancy	Fear of Premature Birth	Pregnancy threat	
I am very afraid of premature birth, I say to myself, lest water sac rupture, lest I bleed, the baby will be born soon				
My brother-in-law's wife, my sister-in-law, and my brother's wife had a history of abortion, and I was also afraid of having an abortion, so for the first three months of my pregnancy, I was only at home and I was only praying that my child would not miscarry	Fear of having a history of recurrent abortion and repeating them	Fear of Abortion		
… I have a history of two miscarriages, one of which had no heart, and now I am all stressed to get to the 18th week of pregnancy and go through it safely				

### Trustworthiness

In the present study, in order to evaluate the accuracy and the quality of the findings, Guba and Lincoln’s four criteria of trustworthiness were used including credibility, dependability, confirmability, and transferability [40]. In this study to ensure credibility, different methods such as strategies of long-term engagement, audio recording, verbatim implementation, member review of codes, peer review, and sampling until data saturation were used as appropriate were used to establish validity. Extracted codes from the five interviews were provided to research associates, study advisors, supervisors, and several other observers, who reanalyzed the interviews. To ensure the dependability of the study results, the methods used to encode the concepts and themes, as well as textual and audio data were attainable. To ensure the confirmability of the data, two experts in qualitative research reviewed the codes and categories obtained from the interviews, and to increase transferability, a clear description of the context, profile, and process of participant selection, data collection, and analysis is provided so that other researchers can decide whether the findings are transferable to their setting or not.

#### Participants

A total twenty- five interviews were conducted with participants, fourteen of whom were pregnant, and eleven of whom were in the postpartum period. Due to pandemic conditions the majority of interviews (90.5%) were conducted by phone. As explained earlier, in order to increase the maximum diversity and benefit from experiences, women who had a history of corona disease also participated in this study, so the conditions in terms of corona disease in the group of pregnant women and the group of mothers after childbirth were as follows.

In the pregnant group: four people were infected with corona disease and in quarantine, four of them had a history of coronavirus during their recent pregnancy but were healthy at the time of the interview, and two of them had a history of infection before recent pregnancy, and four were in good health.

Also, in the postpartum group, three of the mothers were infected with coronavirus during postpartum, two of them were infected with coronavirus during the recent pregnancy and the rest were in perfect health.

The demographic and obstetric characteristics of the participants are shown in Tables 3, 4.

**Table 3:** Demographic characteristics of Pregnant women in the study

Demographic characteristic	Number of participants(%)
Age(year)	
Under 20	1 (7.14)
20–24	3 (21.43)
25–29	3 (21.43)
30–34	5 (35.71)
35–39	2 (14.29)
Education	
Under Diploma	3 (21.43)
Diploma to Bachelor	6 (42.86)
Mas and higher	5 (35.71)
Occupation	
Housewife	9 (64.29)
Employee	5 (35.71)
Obstetric History	
Gravid 1	8 (57.13)
Gravida 2	4 (28.58)
Gravida 3	2 (14.29)
Place of residence	
City	14 (100)
Village	0 (000)
History of other disease	
Yes	2 (14.29)
No	12 (85.71)

**Table 4:** Demographic characteristics of mothers in the study

Demographic characteristic	Number of participants(%)
Age(year)	
20–24	2 (20)
25–29	1 (16)
30–34	4 (36)
35–39	4 (24)
Education	
Under Diploma	2 (11.9)
Diploma to Bachelor	5 (76.2)
Mas and higher	3 (9.5)
University student	1 (2.4)
Occupation	
Housewife	8 (73.8)
Employee	3 (26.2)
Obstetric History	
1 children	4 (36.36)
2 Children	6 (54.54)
3 Children	1 (9.1)
Place of residence	
City	11 (100)
Village	0 (000)
History of other disease	
Yes	7 (63.63)
No	4 (36.37)

## Results

Simultaneous and continuous data analysis led to the extraction of 23 subcategories, 11 categories, and two themes by performing thematic analysis: (1) Maternal mental health threats, (2) Inadequate access to the required information.

The themes and categories are shown in Table 5.

**Table 5:** Themes and categories of current study

Themes	Categories
Maternal mental health threats	Fear of death,
	Parturition threat
	Uncertainty about the neonate's health
	Pregnancy threat
	The stress of neonate care
	Fear of getting coronavirus disease
	The threat of motherly feeling
	Not receiving support
Inadequate access to the required information	Lack of information and knowledge about the fetus/newborn
	Lack of information and knowledge about pregnancy and childbirth
	Lack of information and knowledge about COVID-19 disease

### Main themes

#### Main theme 1: Maternal mental health threats

The participants experienced various worries and fears during this period, and these worries and fears have affected maternal mental health during the COVID-19 pandemic. This theme consisted of eight categories as follows: Fear of death, Parturition threat, Uncertainty about the neonate's health, Pregnancy threat, The stress of neonate care, Fear of catching coronavirus disease, The threat of motherhood sense, and Expect support.

##### Fear of death

This category was the most important that most of the participants expressed. Some of them were very feared of Corona disease. One of these reasons was the daily news of the COVID-19 disease and the number of infected patients, hospitalizations, and deaths, which is broadcast every day on the mass media. Because they thought that due to pregnancy and postpartum conditions, their bodies are not able to withstand the disease and they will definitely die if they get COVID-19 disease. A mother who was infected with Coronavirus in the last pregnancy described her fear of death and concerns said: *“I got corona in the 38th week, my contractions started ten days after the infection, and because my previous delivery was a cesarean section when I was going to the operating room for a cesarean section, I was very scared, I mean you cannot believe, my husband was out of the operating room, and when I entered the operating room, I turned and looked at him again, because I was very disappointed I thought to myself that I might never make it out of the operating room alive, so I looked at him again”* (1*G 3P3*, RC/S, *the forty-fourth day postpartum*, *PP8).*

A pregnant woman who was infected with COVID-19 disease in the 22nd week of her pregnancy stated as follows: *"The news says that if you get corona, you will die. TV, radio, internet all say you will die, you will die I am afraid that I will die too, for example, a nurse who was in the seventh month of pregnancy got coronavirus and died” (G1P0, GA 33*^+*6 day*^*, P2).*

It is almost possible that all or most pregnant women are worried to various degrees about the loss of the fetus during pregnancy and even the death of the baby after delivery, which has various reasons, but a new fear has been added to the worries of mothers during this pandemic, and that is the fear of death the fetus/baby was caused by the coronavirus disease.

One of the pregnant women who was infected with the coronavirus in the 18th week of pregnancy described her concern about the loss of the fetus due to the corona disease as follows:

“Because I was infected with Corona, I was afraid that my baby would die in my womb, I wished that if something happened to her, I would die too and not be alive” (G1P0, GA29 weeks, P8).

The presence of a previous history of fetal death in oneself or in family and friends was another reason for mothers to worry about fetal death.

A healthy pregnant woman at 34 gestational age said "*It happened to one of my colleagues that the umbilical cord was wrapped around the neck of the fetus and the fetus died. I am constantly worried that this will happen to me, and the umbilical cord will wrap around my fetus, and my fetus will die, or perhaps my fetus does not survive after birth! These concerns occupied my mind” (G3P1A1*, *P11).*

##### Parturition threat

Most participants had fears about parturition processes, which were perceived in both kinds of childbirth routes (vaginal, cesarean section) although in the vaginal group, it was greater.

Some participants felt that they lost the ability to normal vaginal delivery due to getting COVID-19 disease*. “I don't know, but I feel sick since I got the coronavirus, I feel like I can't give birth naturally” (G2P1, GA 36 week, P1).*

##### Uncertainty about the neonate's health

The concern for the health of the baby was the first and most important mental concern of mothers.

A woman in the 18th week of pregnancy and with a history of contracting covid-19 disease during pregnancy expressed her concern as follows: *“The concern of all parents is that the child will be healthy and be born healthy, and now that there is the corona, this concern has increased 10 times. My only wish and concern is that my child is healthy and that's all! I have no other worries” (G1P0, P3).*

A 36-year-old mother in 32-week pregnancy and a 4-year-old child said:* “My main concern is that my baby is going to be born healthy without any problems” (G2P1, P4).*

##### Pregnancy threat

Preterm birth and abortion were the other concerns among participants, both healthy and coronavirus-infected ones.

Women who were infected with the coronavirus during pregnancy were much more worried about the occurrence of premature birth due to the infection of coronavirus than healthy pregnant women.

A pregnant mother Infected with covid-19 and in quarantine said: “…. It is said that people who become infected with coronavirus during pregnancy give birth prematurely…. I am worried that I will give birth prematurely due to the coronavirus*” (G2P1, GA 31 week, P14).*

##### The stress of neonate care

Successful breastfeeding was one of the participants' concerns. Most of them were worried and had doubts about having successful breastfeeding, especially nulliparous women.

A nulligravida woman with a gestational age 26-week and with a history of COVID-19 disease during pregnancy said: “*I am pregnant for the first time and I have no experience, so I have very stressed about breastfeeding my baby after birth" (P6).*

Baby care, especially in the early days of birth, was another concern.

A mother who was on her fiftieth postpartum day described her concern about baby care as follows*: “This is my first baby and I didn't know anything about baby care…. The whole family helps me with baby care. For example, I didn't know how to breastfeed, or how to hug the baby, and at the beginning of birth when the baby cried, my whole face was wet from stress” (G1P1, C/S, PP10).*

##### Fear of getting coronavirus disease

The fear of catching/re-infecting the coronavirus was abundantly expressed by the participants. A pregnant woman in the 7th week of pregnancy with a history of corona disease before pregnancy described her concern like this: *“I'm very, very scared, I'm talking about it every day, I'm afraid of catching the coronavirus because I was infected before I got pregnant and my condition was very bad, and because of this, I am very afraid that I will get infected again during pregnancy” (G1P0, P13).*

They were also worried about the effects of COVID-19 disease on themselves/their baby. A mother who was infected with COVID-19 and her baby was also infected after birth said: *“My baby was not well; he is not drinking milk. I was very afraid that we would have to be admitted to the hospital, but thank God, the risk was removed” (G2P2*, *on the twentieth day postpartum*, *RC/S*, *PP11).*

Almost all participants were concerned about intrauterine infection of the fetus or infection of the neonate during or after delivery with coronavirus.

A nulligravida woman in the 37th week of pregnancy described her concern “*… I am very afraid that the child will be infected! Because now they say that it is possible for children to catch coronavirus in their mother's womb, I'm afraid that my baby gets infected….*” *(P5).*

##### The threat of motherly feeling

Although pregnancy is one of the best times of a woman's life, not everyone who gets pregnant necessarily feels good. Some participants expressed regret that they became pregnant during the COVID-19 pandemic.

A nulligravida woman in the 18th week of pregnancy described her feeling about being pregnant during the pandemic as this: “… On the other hand, we have to stay in the house all the time. A person gets depressed. This thing is very annoying. My thoughts are really fluctuating, I tell to myself, wow, now it was not the time to be pregnant, I wish I had acted a little earlier, I wish there was no corona” (P12).

In light of the pandemic and ensuing quarantine measures and closure and the reduction of family visits, many participants have reported feeling lonely and isolated, this is particularly true for those who have contracted COVID-19, because they were very worried about their health and their fetus/baby, and on the one hand, they felt lonely and the absence of people around them made their mental conditions much more complicated, although the severity of the change was not the same for everyone.

A woman in the 36th week of pregnancy, suffering from corona disease, who was in quarantine, described her mental condition as follows: *“I am very sad in this quarantine, very sad; It's been ten days now that I haven't seen my mother because I was infected with Corona. I'm very depressed. I miss my parents and my brother very much” (G2P1, P1).*

##### Not receiving support

During pregnancy, and after childbirth, women are emotionally and physically sensitive. Their minds are involved in many worries and questions about the baby's future, the baby's health, relationship, etc. Receiving support, especially from spouse and family, and friends can make mothers feel better. In fact, mothers expect more support from their husbands and those around them during this period than at other times.

A mother with a third pregnancy who was 34 weeks pregnant expressed her expectations from her husband like this: “… *He doesn't support me at all, I expect him to support me mentally, emotionally, helping me with housework and… But he does not support me. I tell him you lied to me, you said if you get pregnant I will support you, and unfortunately, I did not receive any support*” *(G3P1A1, P11).*

In addition to spouse support, some participants expected and needed the support of family during pregnancy and after delivery.

“I expect my sister, who does not have a small child, to come and help me” (G3P3, On the fifth day of postpartum, PP9).

#### Main theme 2: Inadequate access to the required information

Another factor causing concern among the participants was inadequate access to the required information. Most of them, especially nulliparous and new mothers, believed that they don't have any information about pregnancy, childbirth, postpartum, baby care, and also about corona disease, and due to the pandemic situation, many of their questions remain unanswered (Due to the fear of getting infected when visiting health centers, reducing the duration of visits, and etc.). on the other hand, there is a lot of fake information, especially about COVID-19, especially in cyberspace, which has doubled their worries.

This theme consisted of three categories as follows: Lack of  information and knowledge about the fetus/ neonate, Lack of information and knowledge about pregnancy and childbirth, and Lack of information and knowledge about COVID-19 disease.

##### Lack of information and knowledge about the fetus/newborn

The participants said that they needed information about the fetus/baby, especially, nulliparous and new mothers. They needed to gain information and knowledge about the growth and development of the fetus in the uterus, fetal movement, proper nutrition for fetal growth, intelligence, various risk factors affecting the health of the fetus, neonate care after birth, vaccination and etc.

A 39-year-old woman in the 13th week of pregnancy said: *“I have no information at all about the fetus, I have no information, and every information about the fetus is good for me*” *(G2P0A1, P7).*

As explained above, in addition to information about the fetal period, they also needed information about the neonatal period, education about neonate jaundice, breastfeeding, umbilical cord care, weight gain, etc. among the informational needs of mothers after childbirth.

A pregnant woman with COVID-19 disease said: *"… What should I eat that is good for him? What should I do to make it good for my child? What position should I sleep in so as not to harm my baby? What should I eat to prevent jaundice after birth?” (G2P1, GA 7 week,* in quarantine*, P10).*

A mother who delivered twelve hours ago *said: “What should I eat now that won't harm the baby? What should I eat to prevent neonate jaundice? What should I do to make him smart? What should I do to better grow” (G2P2, RC/S, PP5).*

##### Lack of information and knowledge about pregnancy and childbirth

The participants stated that due to the conditions of the COVID-19 pandemic, many of their questions remained unanswered and they needed a lot of information about pregnancy, childbirth, and the postpartum period. Due to the pandemic situation, face-to-face childbirth preparation classes have been stopped and tele classes have replaced them, but due to access problems, these types of communication have not been as successful as face-to-face classes.

Most of the participants, especially nulliparous, were worried about childbirth and needed information about appropriate activities, and exercises to prepare for childbirth. A pregnant woman with COVID-19 disease said: “*I would like to know what sports I should do and what I should do to make my birth easier with less pain, as well as I would like to know what could I myself do to become more prepared for childbirth” (*G2P1, GA31  weeks, in quarantine, *P14).*

##### Lack of information and knowledge about COVID-19 disease

There was a strong need to be aware of COVID-19 disease and its consequences for the fetus/infant, the mother, and those around them during the pandemic. The larger part of the participants expressed a strong demand to learn about the potential consequences of contracting the virus, not only for themselves but also for their families, especially for their neonate even in future years. Unfortunately, the lack of accurate information and knowledge on this topic greatly had been caused anxiety and worry among them.

A pregnant woman who had a history of COVID-19 disease before pregnancy said: *“I really don't know what will happen to me if I get corona now that I am pregnant? What effect will it have on my baby? I'm stressed because I don't know, If I know what effects it will have on my child if I get Corona, my stress will be reduced! Is it possible, for example, to damage the child's nervous system or growth? I am looking for definitive answers to these questions” (G1P0, GA 18Weeks, P12).*

A pregnant woman who suffered from COVID-19 disease asked:*”What kind of food should I eat that is suitable for me now that I have corona disease? What foods are prohibited in Corona?” (G3P1A1, 17 weeks*, in quarantine, *P9).*

A mother who, on the twelfth day after birth, everyone in her family, even her baby, was infected with COVID-19 disease said: “Does *it really give immunity for 5–6 months? Is it true? When can I get corona vaccine? What effect does it have on my child? Don't have complications later” (G3P3, On the fifth day of postpartum, PP9).*

## Discussion

This article provides an in-depth insight into the mental health needs of mothers in Iran during the COVID-19 pandemic, which is presented for the first time. The results showed that mothers in addition to the typical worries related to pregnancy and postpartum experienced a lot of worries and anxieties during this time. Fear was the most common term used by them to describe mental health concerns during the pandemic, such as fear of death, fear of infection, fear of premature birth, fear of infant infection, etc. They had also been burdened by stress and confusion caused by the unprecedented circumstances of the pandemic. These feelings and concerns were caused by the perception of the great severity of the threat and the sense of uncertainty about the health and survival of the own, family, especially the fetus/baby, the unknown nature of the disease, the high mortality rate, and its unidentified effects on pregnancy, childbirth, and postpartum.

The findings of theme 1 indicated that the mental health of pregnant and postpartum women was affected by the pandemic. These threats can disrupt pregnancy and postpartum health and lead to abnormal experiences. Finally, they may cause complications in the short and long term for mothers, children, and families [41–49]. The main concern was the fear of own death or the death of the baby/fetus due to corona disease, which could be caused by the high lethality of this pandemic and the feeling of vulnerability caused by pregnancy. Almost all infected mothers and most of the healthy mothers expressed their fear of dying due to COVID-19. Ahorsu et al. in evaluating the psychometric properties of the scale of fear of COVID-19 concluded that the phrase "I am afraid of losing my life due to the coronavirus" had the highest factor loading [50]. Fear of death has also been found in other studies [51, 52]. The pandemic was a traumatic experience because it had a threatening nature and the resulting deaths had a negative impact on people's mental health [53]. The illness or death of friends or relatives due to the disease of COVID-19 was another stressor of COVID-19 in this study. The "infodemic" (a mass of information and sad news about COVID-19) by various sources and social media [54], was also another source of fear and anxiety among the participants. According to studies the anxiety and worries created by media were more than the awareness provided to prepare against this pandemic [30, 55–57]. Limaye et al. claimed that the spread of misinformation about COVID-19 was facilitated by social media platforms [58]. As our study spanned from the conclusion of the initial wave to the midpoint of the subsequent wave, perhaps one reason for participants' fears is the severity of the pandemic, as well as a significant number of individuals who had been infected or had passed away. This result was in line with a study in Brazil [59]. Another was due to novelty and uncertainty about the pandemic process which has been emphasized in other studies [24, 60]. The newborn’s health was another important concern, for all participants. In fact, one of the essential reasons for the heightened anxiety of mothers regarding COVID-19 was the impact on the health of their unborn or newborn child. All of them, without exception, believed that their infection with COVID-19 will result in unsafe results such as intrauterine disease of the fetus or infection of the baby after delivery in the hospital and damage to the child's lungs. Consistent with our study, Barbosa-Leiker et al. showed that the fear of getting COVID-19 the baby in the hospital was one of the concerns expressed by pregnant and post-partum women (up to one year after delivery) in the USA [61]. Many individuals in our study expressed concern regarding their health, specifically in relation to potential lung injury caused by infection. Those who had a history of COVID-19 or knew somebody who did, experienced a higher level of fear and anxiety than the possibility of re-infection. This was in line with the studies conducted in Jordan, the UK, China, Canada, and Germany [58, 62–65]. In our study, most of the participants even healthy mothers with no history of previous pregnancy complications were worried about the occurrence of pregnancy complications, such as premature birth and abortion. Of course, those who had a history of such complications in previous pregnancies or in their relatives were more worried. Other studies showed that COVID-19 causes an increase in some pregnancy complications such as premature labor and abortion [66–68]. One of the reasons for this is the unknown nature of the COVID-19 disease and its complications, and the other reason is perhaps talking with peers and hearing the experiences of those who had a history of these complications. This finding is in line with other studies [69, 70]. The participants in our study were forced to socially distance themselves from their friends and family due to the fear of contracting COVID-19 and this caused them to feel lonelier and lack of support. The absence of family and Loneliness during childbirth were other concerns of moms. Isolation appears to have had a deep effect on the participants, which highlighted the need for adequate support at the stages of pregnancy and postpartum. Loneliness and social distancing, which involves contact with loved ones and friends, increase stress, anxiety, and depression and have been reported by pregnant and postpartum women in some studies [71–76]. Since pregnant and postpartum women are more vulnerable to the negative consequences of loneliness and isolation [77, 78], it is necessary to pay attention to the provision of social support especially in critical situations such as the recent pandemic [64, 79]. Maternal care providers can play a role to reduce these negative effects by offering various ways such as counseling, prenatal yoga, and birth classes online and others. [80]. Due to the unprecedented circumstances of the COVID-19 pandemic, participants in this study had heightened expectations from healthcare providers. In addition to the standard pregnancy and postpartum care, they required sufficient support and dedicated time for their inquiries. Also, particularly new mothers were anxious about infant healthcare. They needed more support and help from healthcare providers. This result was in line with other studies [81–83]. This highlights the importance of healthcare providers being responsive and more attentive to the needs of participants in times of crisis. In order to prevent possible infection in Iran, like other countries [29, 54, 79, 84, 85], the number of pregnancy and post-natal visits decreased. Face-to-face childbirth preparation classes were closed, and information was available offline, through educational CDs and booklets. Of course, there was a special hotline in the Ministry of Health to respond to the needs of mothers and pregnant women 24 h a day, but nevertheless, participants stated that they should receive education related to pregnancy, childbirth, and postpartum as before. And they wanted the classes to be held face-to-face and with a small number. Those who were infected with COVID-19 expected that during the quarantine period, they would definitely have an in-person pregnancy visit or a daily phone call with the obstetricians because they believed that they were very worried about the health of their fetus. On the other hand, they refused to go to health centers and especially hospitals on time due to the fear of contracting COVID-19. It seems that this paradox may have been due to the concern of the participants about the health of the fetus and the high stress caused by the COVID-19 pandemic. On the one hand, they were worried about the health of their fetus, and on the other hand, they did not go to health and treatment centers due to the fear of the possibility of contracting COVID-19 and endangering the health of the fetus. Because some believed that if they get infected, the fetus will be harmed, or that they themselves will be harmed or even die, and in this case, their child or children will be left without a mother.

The findings of Theme 2 highlighted the importance of paying attention and the availability of accurate information from reliable sources. Due to various factors, such as a decrease in the number of pre and postnatal visits and the closure of in-person pregnancy and childbirth classes and etc. participants had limited interaction with healthcare providers. This resulted in a lack of knowledge and information about COVID-19, as well as pregnancy and postpartum-specific information, which led to participants more experienced stress and worry. Therefore, access to correct information is very useful and vital in a pandemic situation [86].

The results of our research were in line with several studies in different countries such as Ireland, Turkey, Iran, China, and the UK which, from various aspects, identified the deficiencies and information needs of pregnant and postpartum women during the COVID-19 pandemic and its consequences [24, 87–92].

In this study, the Internet and social networks were the dominant media for accessing this information, yet many of them were concerned about the validity of the information. All of them generally wished for more information that was available from valid resources such as healthcare professionals. Researchers from other countries also reached this conclusion in their studies [86, 93, 94]. Therefore, one factor for the great fear of COVID-19 disease is the lack of information and misinformation [60]. Examining and comparing the results of this study with various studies from other countries indicates that the mental health of pregnant women and new mothers has been greatly affected by the direct and indirect consequences of the COVID-19 pandemic. Many quantitative and qualitative studies were from different aspects. In some cases, the problems were common, but in some cases, they were special, which seems to be due to the time to conduct studies, and the cultural, organizational, and economic differences of different countries. Now that the world is almost moving towards the post-pandemic era, it is suggested that policymakers take advantage of the many results of research conducted all over the world to plan various necessary measures to maintain, promote or improve the mental health of this vulnerable group.

## Strengths and limitations

In this study, we tried to have maximum diversity in the participants. They were in the groups with coronavirus disease, recovered, pre-pregnancy coronavirus disease, and spouse infection. Most interviews were conducted by telephone due to pandemic conditions. Therefore, it was not possible to take field notes and observe the participant's behavior and record non-verbal data. Moreover, the saturation of the interview was achieved with more interviews. Due to the spread of the COVID-19 pandemic and the fear of infection, some mothers were reluctant to participate in the study, and some did not go to the health centers for visiting entirely. Consequently, there may be data that has gone unnoticed. Maternal healthcare providers should try to address these concerns in various ways, such as informative yet empathetic messages, psychological, and social support, and others. At the same time, policymakers should design and implement appropriate programs and measures to improve mothers' mental health and prevent negative experiences, especially in critical situations. Hence, it seems that identifying the needs and concerns of mothers in crisis can help policymakers to plan effectively to increase mental health care and support during future epidemics and emergencies. Therefore, the findings of the present study will be useful in planning the promotion of women's mental health during pregnancy and postpartum, especially in critical situations (disaster settings and/or high-risk pregnancy situations). In this regard, this project is suggesting the following items to the National Maternal Health Policymakers to put more stress on maternal mental health well-being:

- Providing a sustainable teleconnection/consultation platform for mothers-to-be.
- Strengthening mental health issues in childbirth preparation training classes.
- Strengthening family support during pregnancy and childbirth and postpartum periods.

Suggestions for future studies:

Based on the study's findings, particularly the explained concerns, it is recommended to create and implement a maternal mental health training program, during the pandemic. Additionally, it is suggested to design and evaluate a tool to measure maternal mental health concerns, using the definition provided by these findings.

## Conclusion

Although much attention has been paid to the physical health consequences of COVID-19, mental health has unfairly received less attention. There is a need to prepare research and healthcare infrastructure to investigate the effects of the COVID-19 pandemic on mental health, which requires proper research, especially for vulnerable groups [37]. Our findings showed that the mental health concerns of women in pregnancy and postpartum during the COVID-19 pandemic are diverse and in the form of mental and emotional problems and information needs that are not met. These concerns, directly and indirectly, significantly impact threatened maternal mental health during the COVID-19 pandemic.

## Data Availability

All data generated or analyzed during this study are included in this published article.

## Footnotes

1 G: Gravida. P: Parturation. A: Abortion. R C/S: Repeated Cesarean Section. PP: Postpartum. GA: Gestational Age.
